# Supplementary figures and images for: Multi-omics insights into host-viral response and pathogenesis in Crimean-Congo hemorrhagic fever viruses for novel therapeutic target
Source: eLife. 2022 Apr 19;11:e76071. doi: 10.7554/eLife.76071 (PMC9018070; doi:10.7554/eLife.76071)

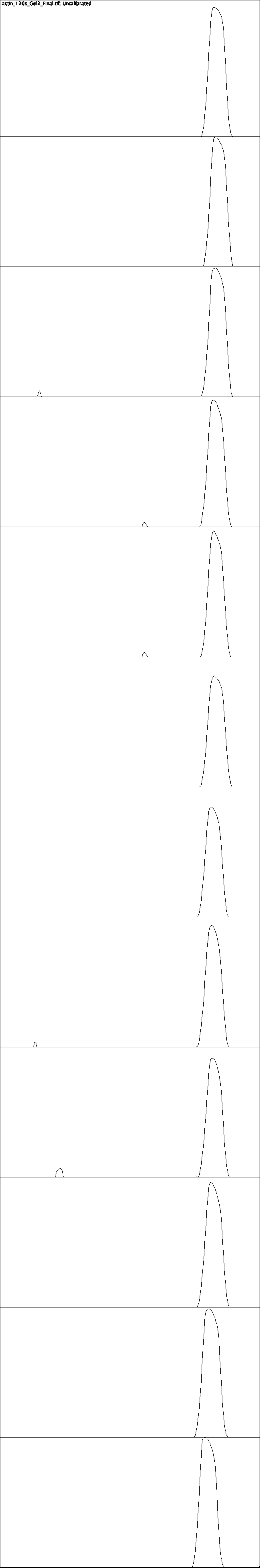

Supplement: Figure 5—source data 1. [file elife-76071-fig5-data1.zip › Source_Data_1/WB_CCHFV_ISG/FIJI Densitometry Plots/R1R2/Plots of actin_120s_Gel2_Final.tif]

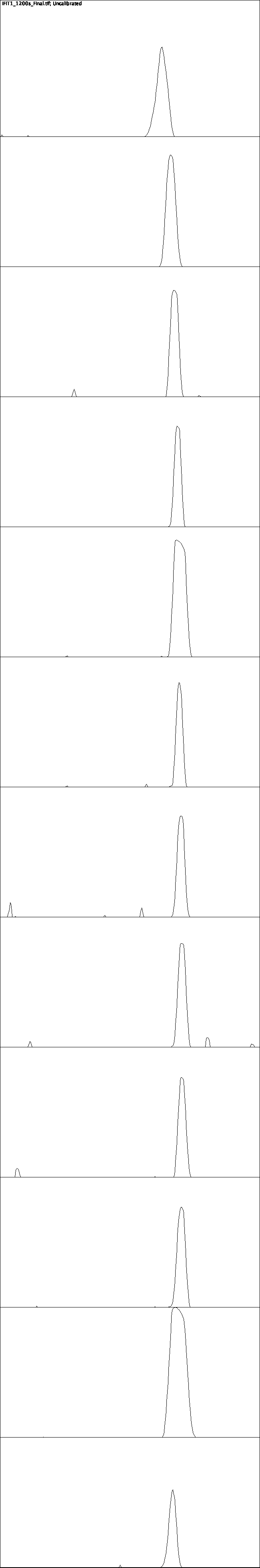

Supplement: Figure 5—source data 1. [file elife-76071-fig5-data1.zip › Source_Data_1/WB_CCHFV_ISG/FIJI Densitometry Plots/R1R2/Plots of IFIT1_1200s_Final.tif]

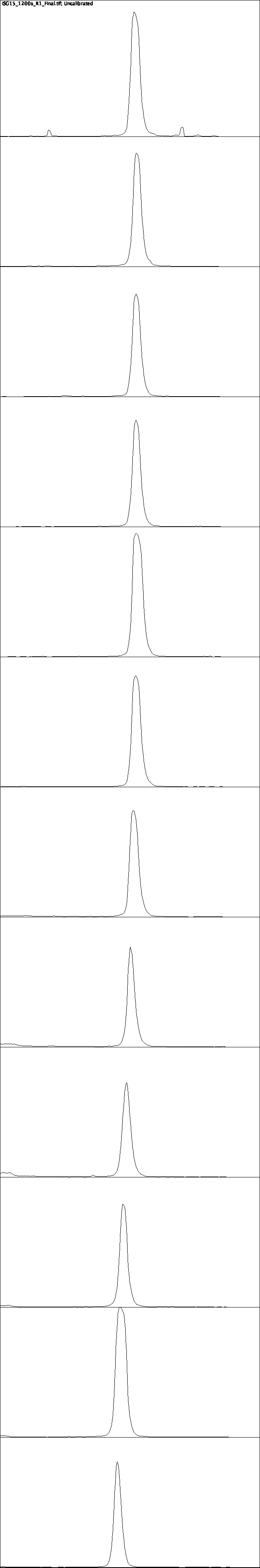

Supplement: Figure 5—source data 1. [file elife-76071-fig5-data1.zip › Source_Data_1/WB_CCHFV_ISG/FIJI Densitometry Plots/R1R2/Plots of ISG15_1200s_R1R2_Final.tif]

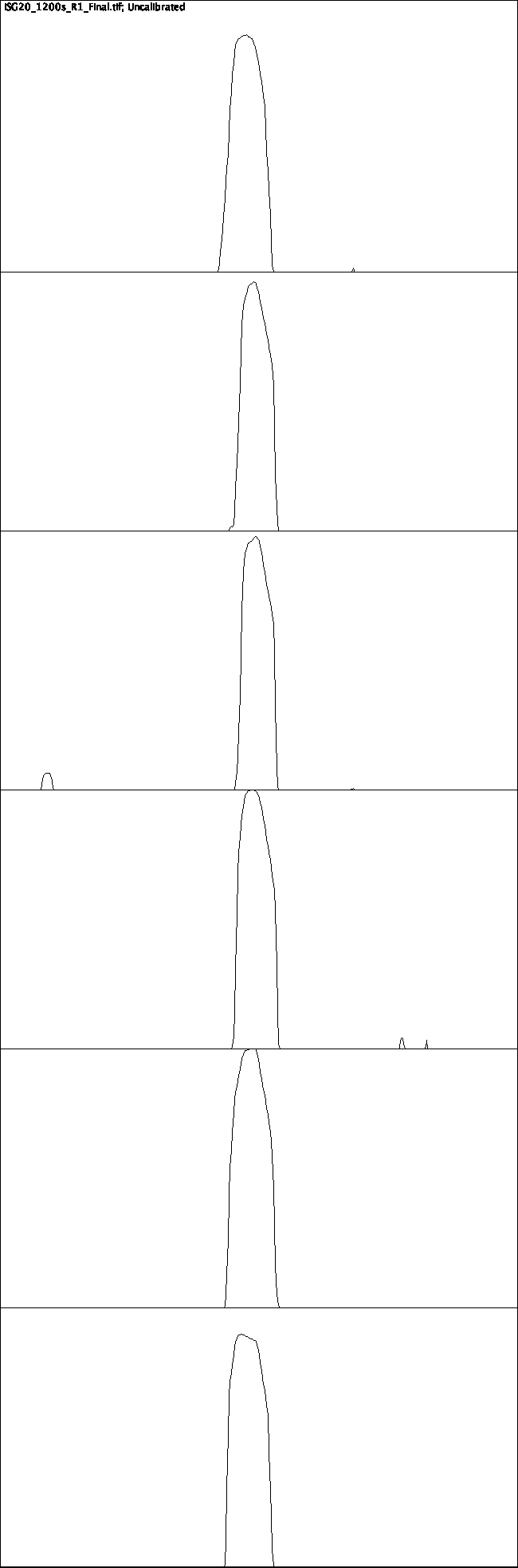

Supplement: Figure 5—source data 1. [file elife-76071-fig5-data1.zip › Source_Data_1/WB_CCHFV_ISG/FIJI Densitometry Plots/R1R2/Plots of ISG20_1200s_R1_Final.tif]

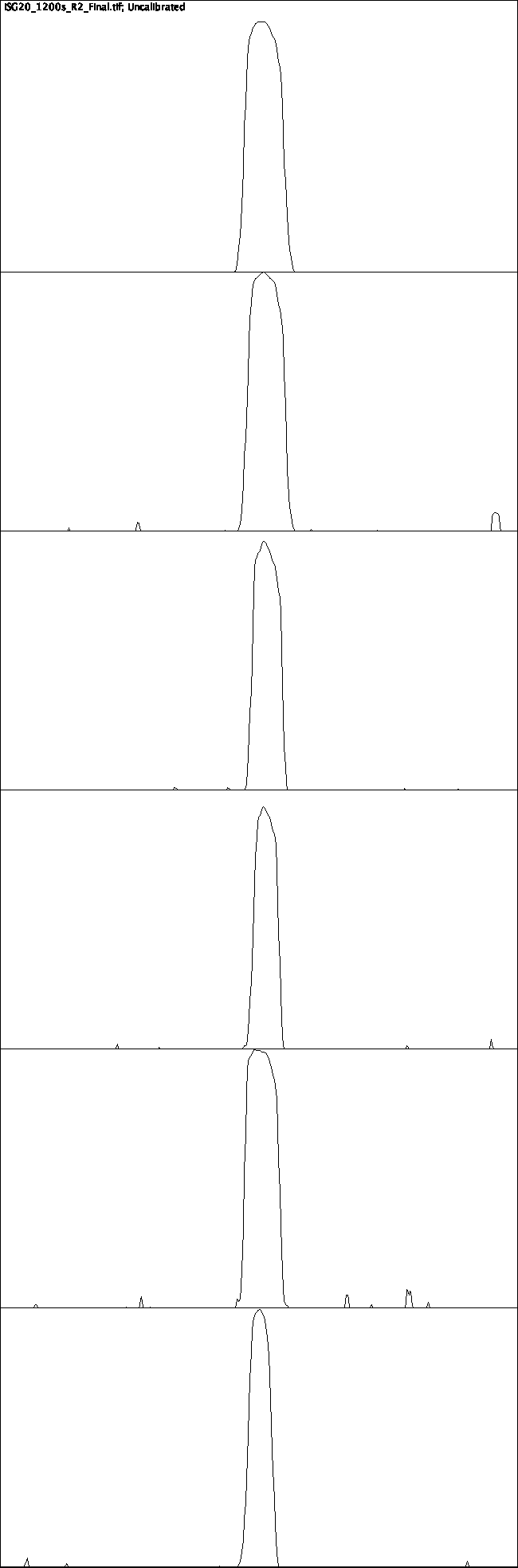

Supplement: Figure 5—source data 1. [file elife-76071-fig5-data1.zip › Source_Data_1/WB_CCHFV_ISG/FIJI Densitometry Plots/R1R2/Plots of ISG20_1200s_R2_Final.tif]

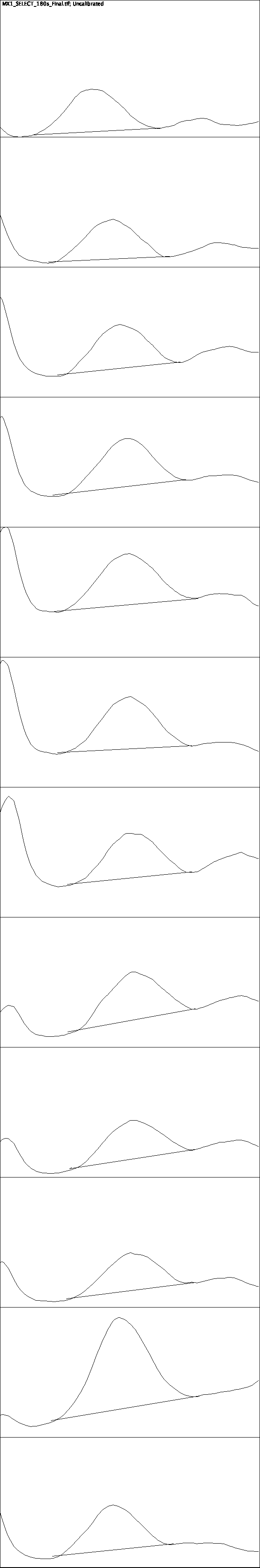

Supplement: Figure 5—source data 1. [file elife-76071-fig5-data1.zip › Source_Data_1/WB_CCHFV_ISG/FIJI Densitometry Plots/R1R2/Plots of MX1_SELECT_180s_Final.tif]

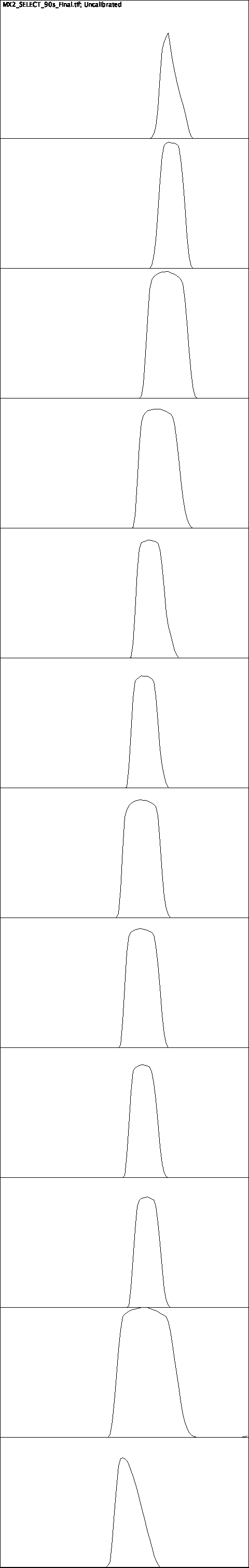

Supplement: Figure 5—source data 1. [file elife-76071-fig5-data1.zip › Source_Data_1/WB_CCHFV_ISG/FIJI Densitometry Plots/R1R2/Plots of MX2_SELECT_90s_Final.tif]

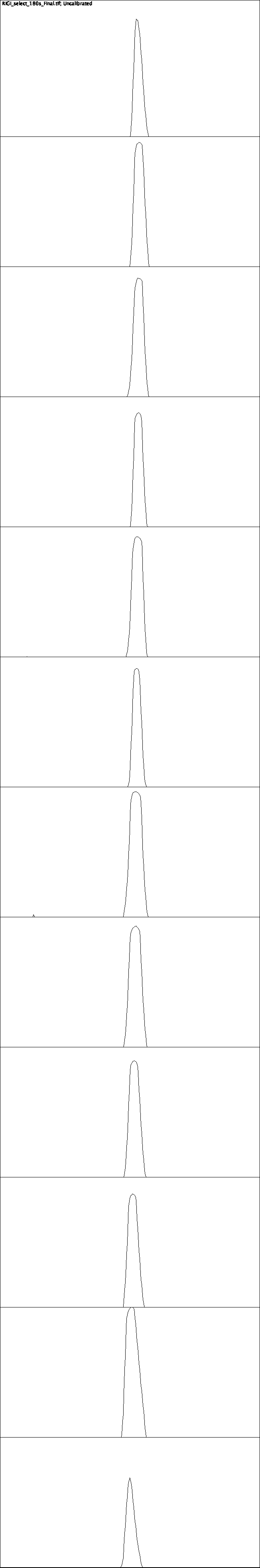

Supplement: Figure 5—source data 1. [file elife-76071-fig5-data1.zip › Source_Data_1/WB_CCHFV_ISG/FIJI Densitometry Plots/R1R2/Plots of RIGI_select_180s_Final.tif]

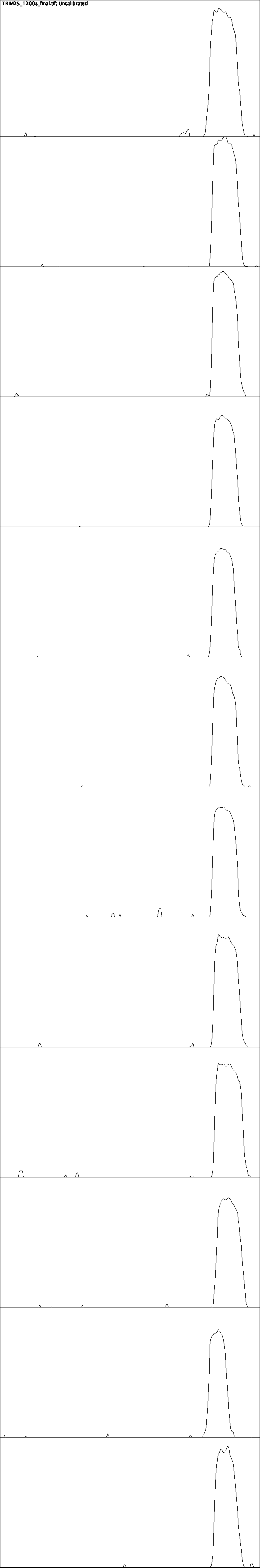

Supplement: Figure 5—source data 1. [file elife-76071-fig5-data1.zip › Source_Data_1/WB_CCHFV_ISG/FIJI Densitometry Plots/R1R2/Plots of TRIM25_1200s_final.tif]

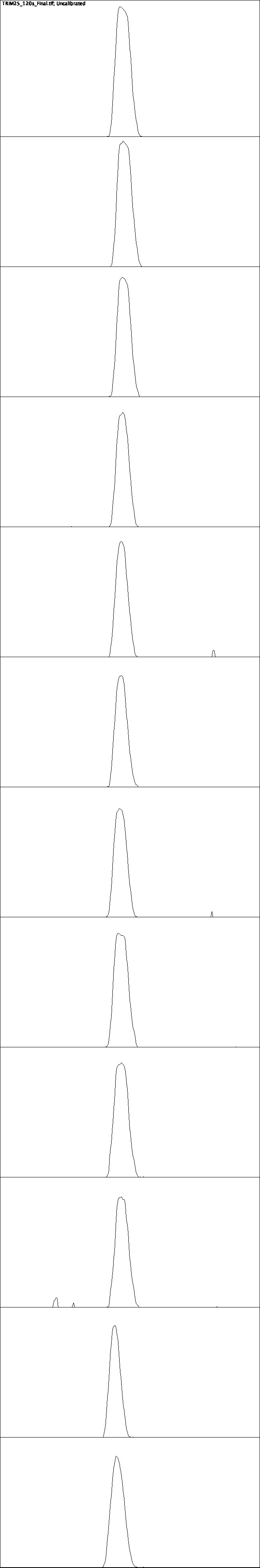

Supplement: Figure 5—source data 1. [file elife-76071-fig5-data1.zip › Source_Data_1/WB_CCHFV_ISG/FIJI Densitometry Plots/R1R2/Plots of TRIM25_120s_Final.tif]

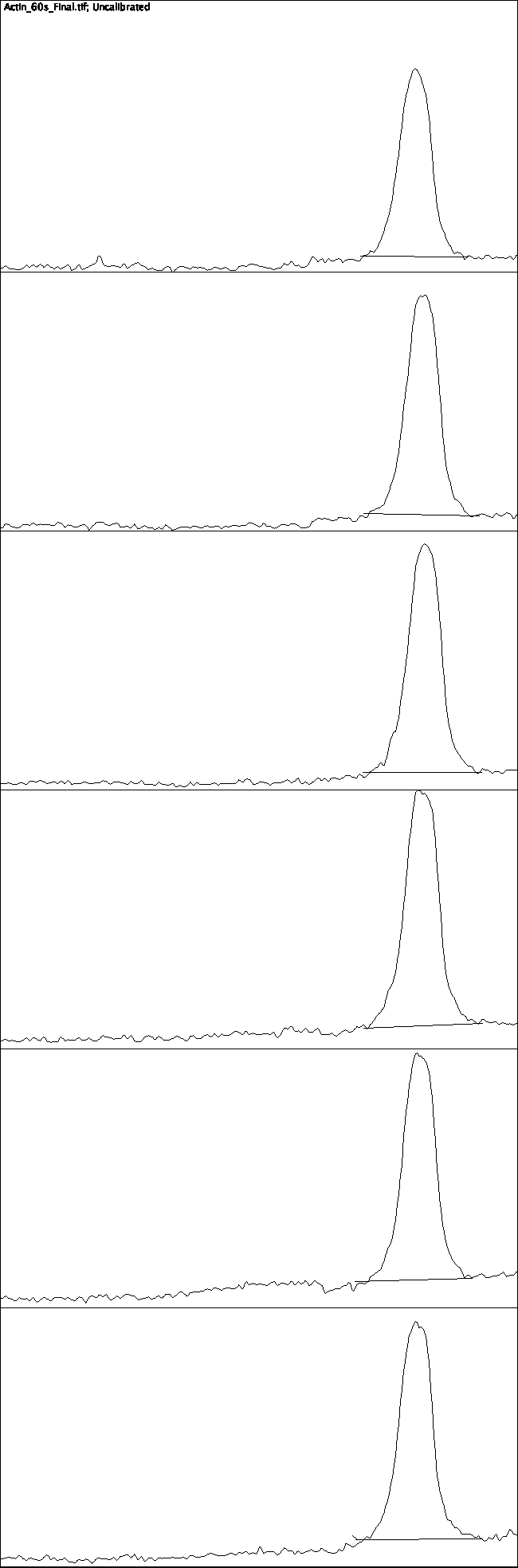

Supplement: Figure 5—source data 1. [file elife-76071-fig5-data1.zip › Source_Data_1/WB_CCHFV_ISG/FIJI Densitometry Plots/R3/Plots of Actin_60s_Final Gel1.tif]

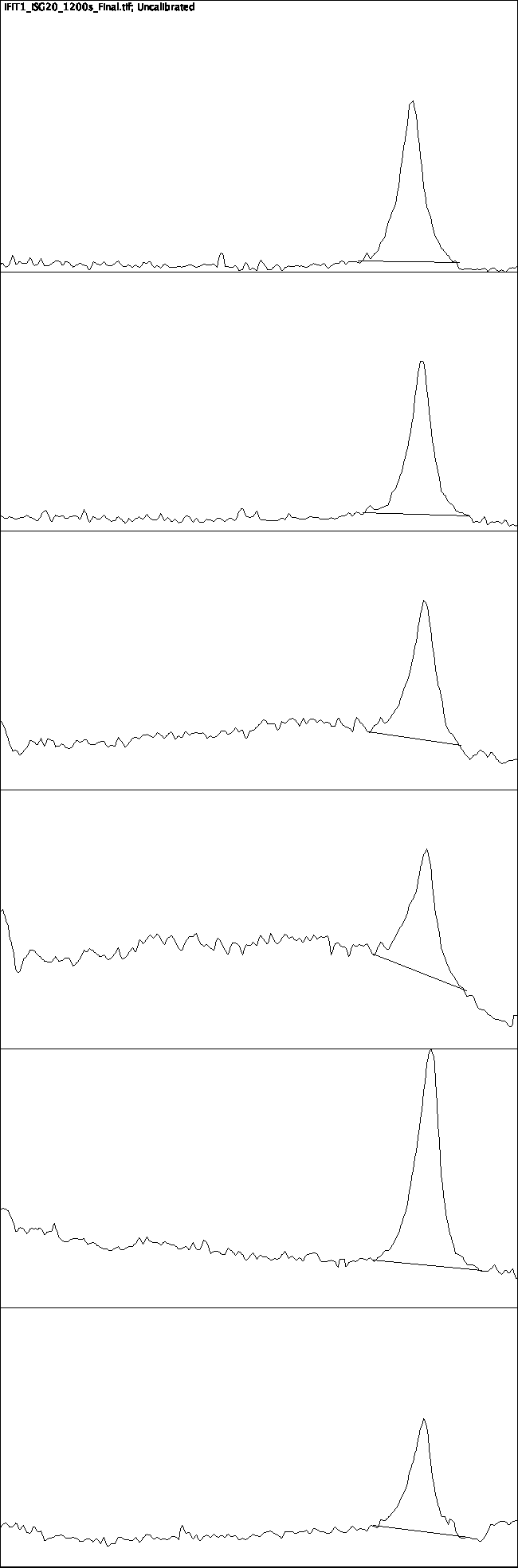

Supplement: Figure 5—source data 1. [file elife-76071-fig5-data1.zip › Source_Data_1/WB_CCHFV_ISG/FIJI Densitometry Plots/R3/Plots of IFIT1_1200s_Final.tif]

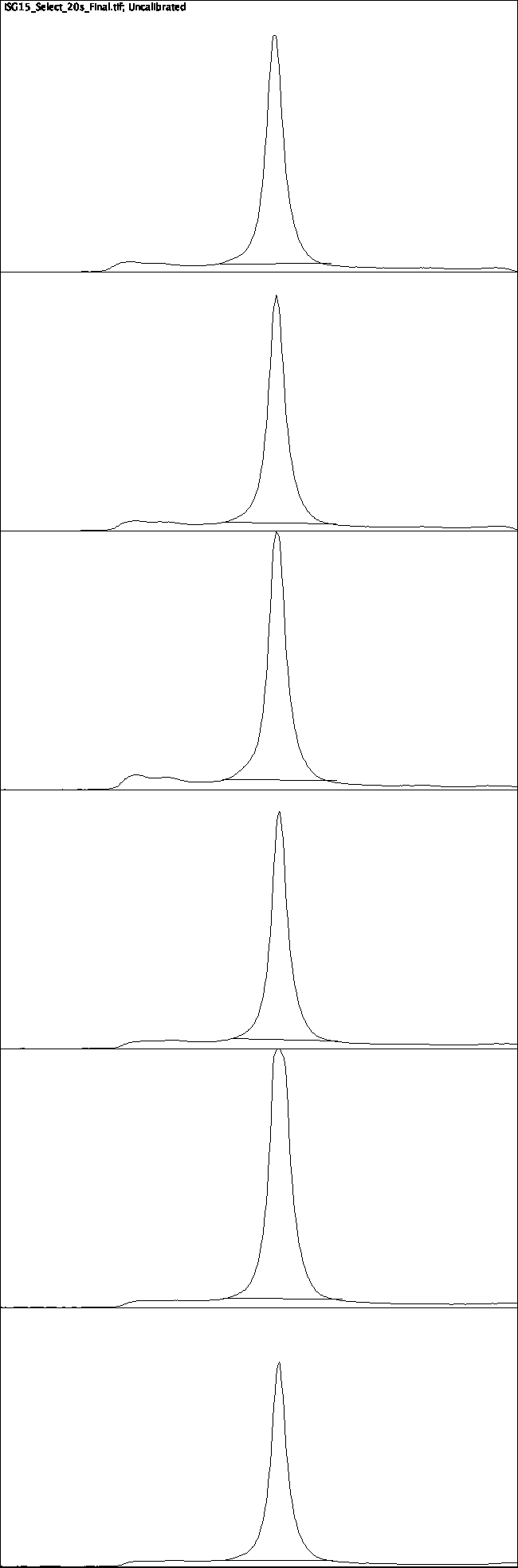

Supplement: Figure 5—source data 1. [file elife-76071-fig5-data1.zip › Source_Data_1/WB_CCHFV_ISG/FIJI Densitometry Plots/R3/Plots of ISG15_Select_20s_Final.tif]

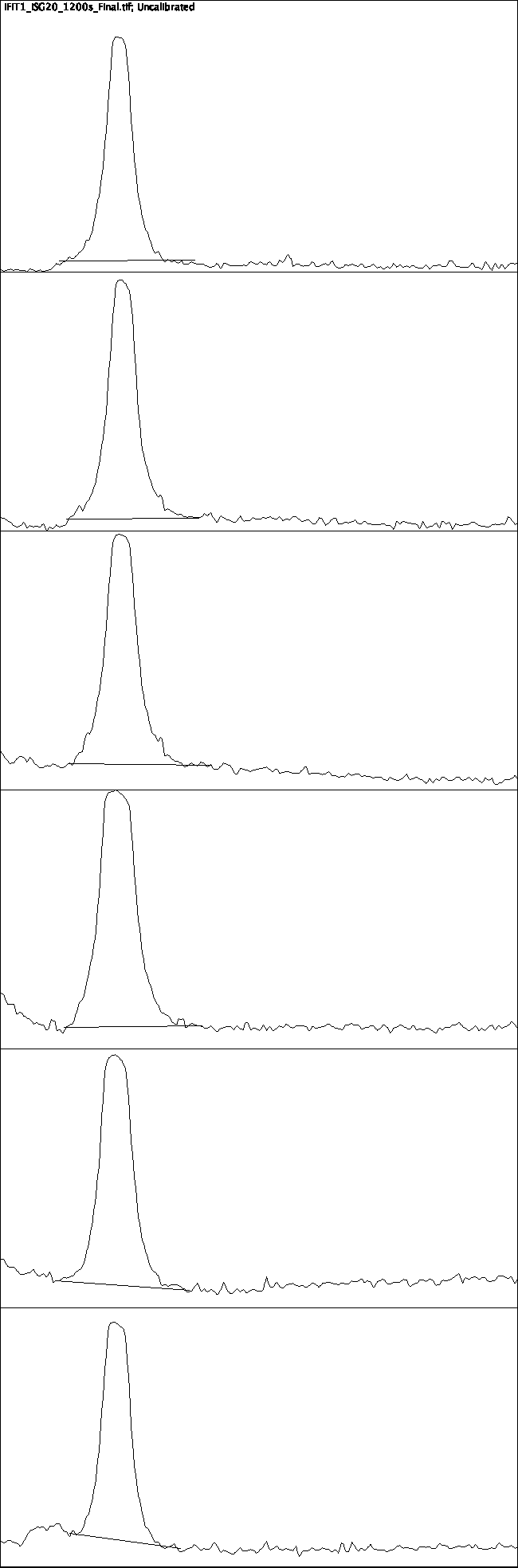

Supplement: Figure 5—source data 1. [file elife-76071-fig5-data1.zip › Source_Data_1/WB_CCHFV_ISG/FIJI Densitometry Plots/R3/Plots of ISG20_1200s_Final.tif]

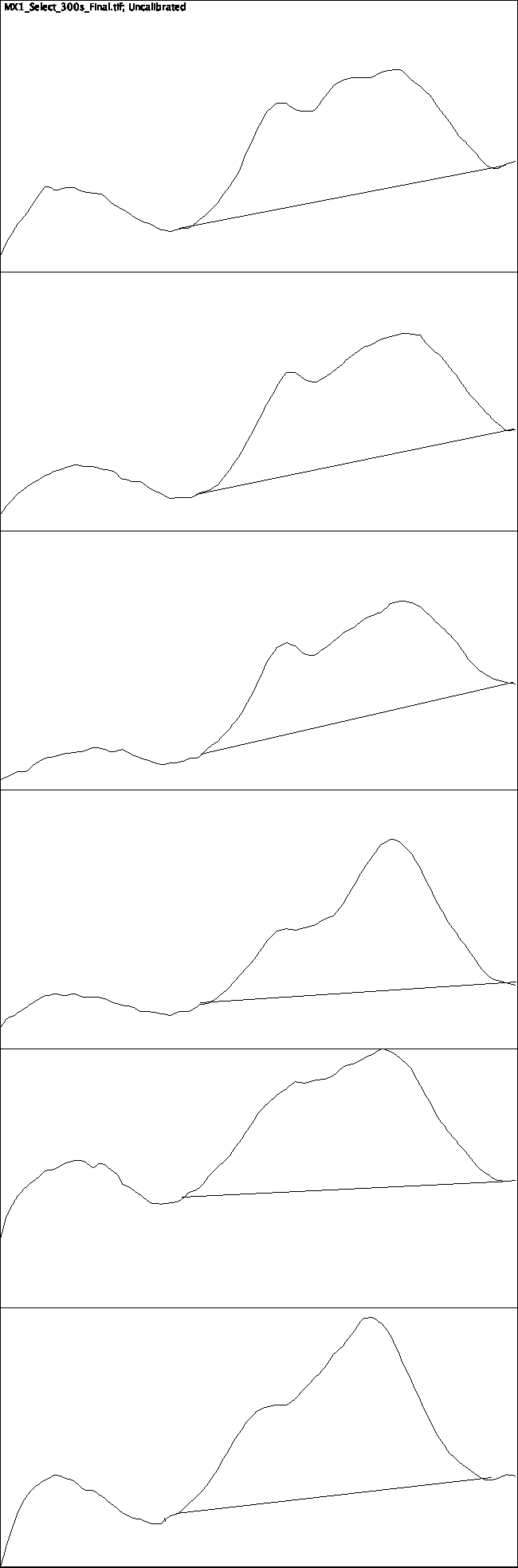

Supplement: Figure 5—source data 1. [file elife-76071-fig5-data1.zip › Source_Data_1/WB_CCHFV_ISG/FIJI Densitometry Plots/R3/Plots of MX1_Select_300s_Final.tif]

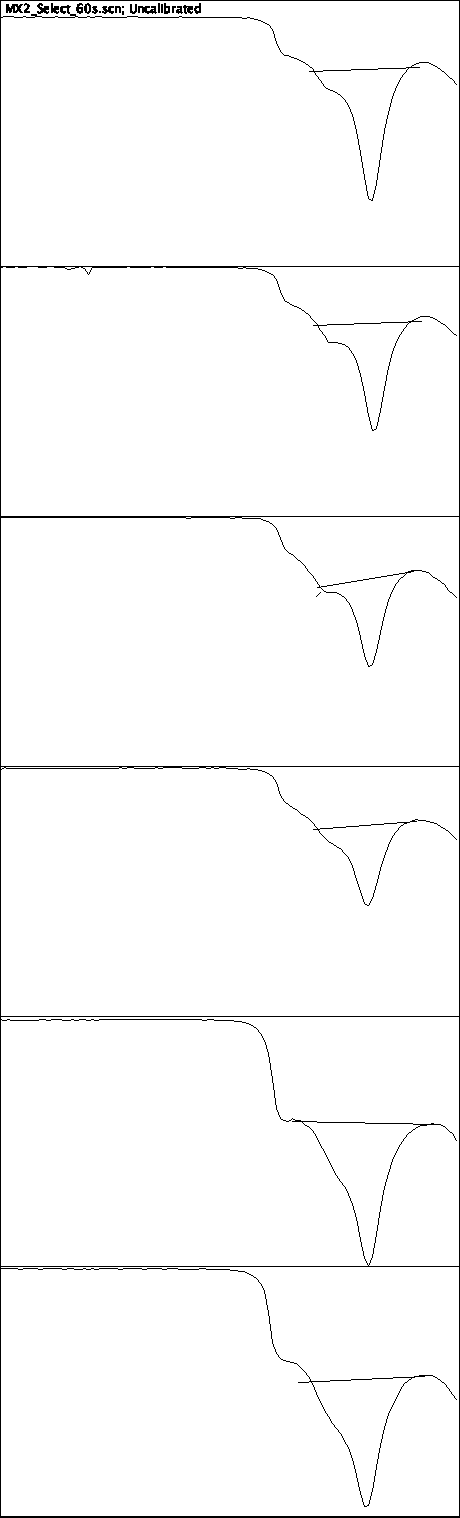

Supplement: Figure 5—source data 1. [file elife-76071-fig5-data1.zip › Source_Data_1/WB_CCHFV_ISG/FIJI Densitometry Plots/R3/Plots of MX2_Select_60s_invert.tif]

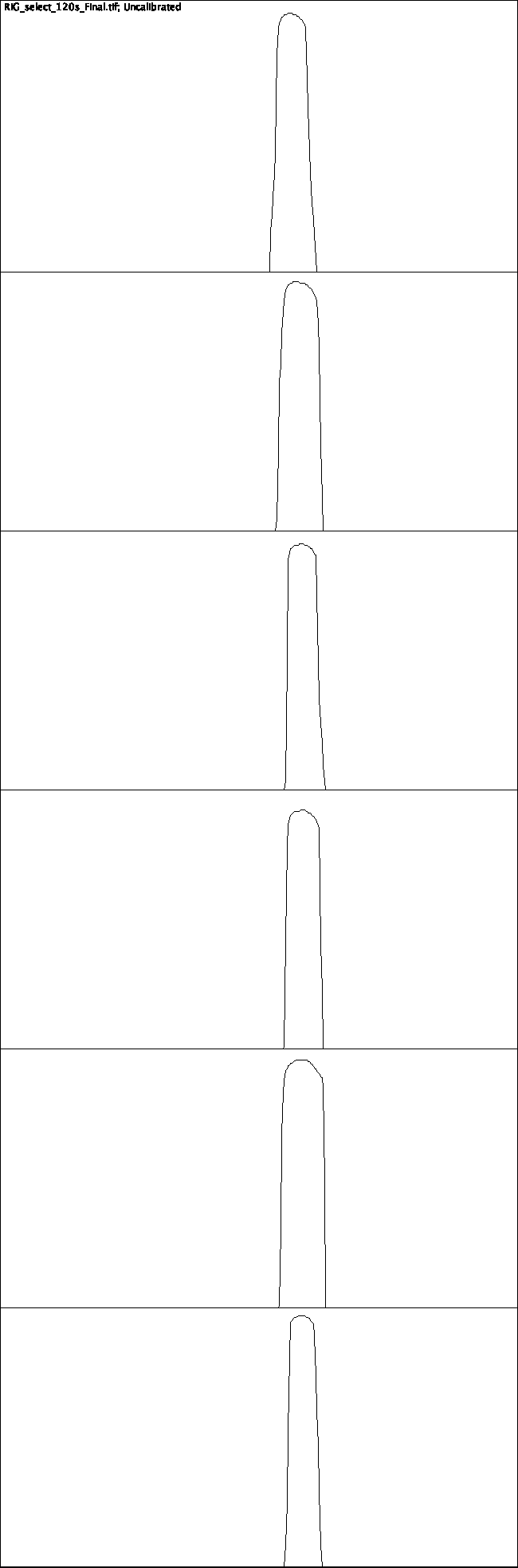

Supplement: Figure 5—source data 1. [file elife-76071-fig5-data1.zip › Source_Data_1/WB_CCHFV_ISG/FIJI Densitometry Plots/R3/Plots of RIG_select_120s_Final.tif]

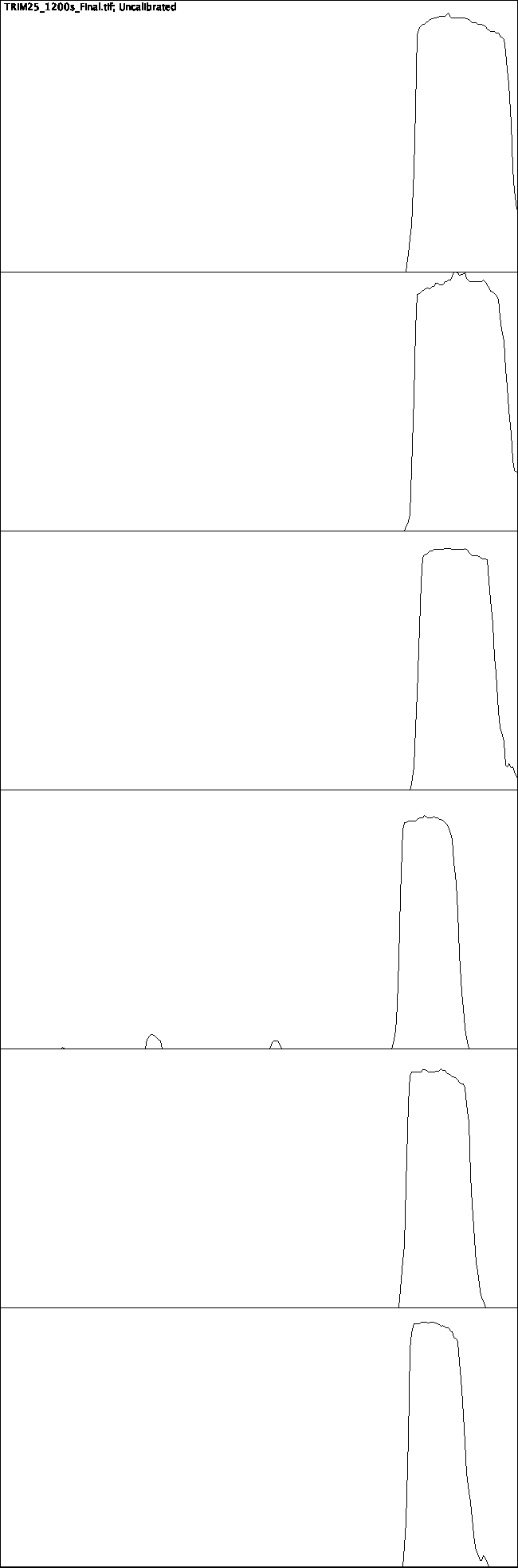

Supplement: Figure 5—source data 1. [file elife-76071-fig5-data1.zip › Source_Data_1/WB_CCHFV_ISG/FIJI Densitometry Plots/R3/Plots of TRIM25_1200s_Final.tif]

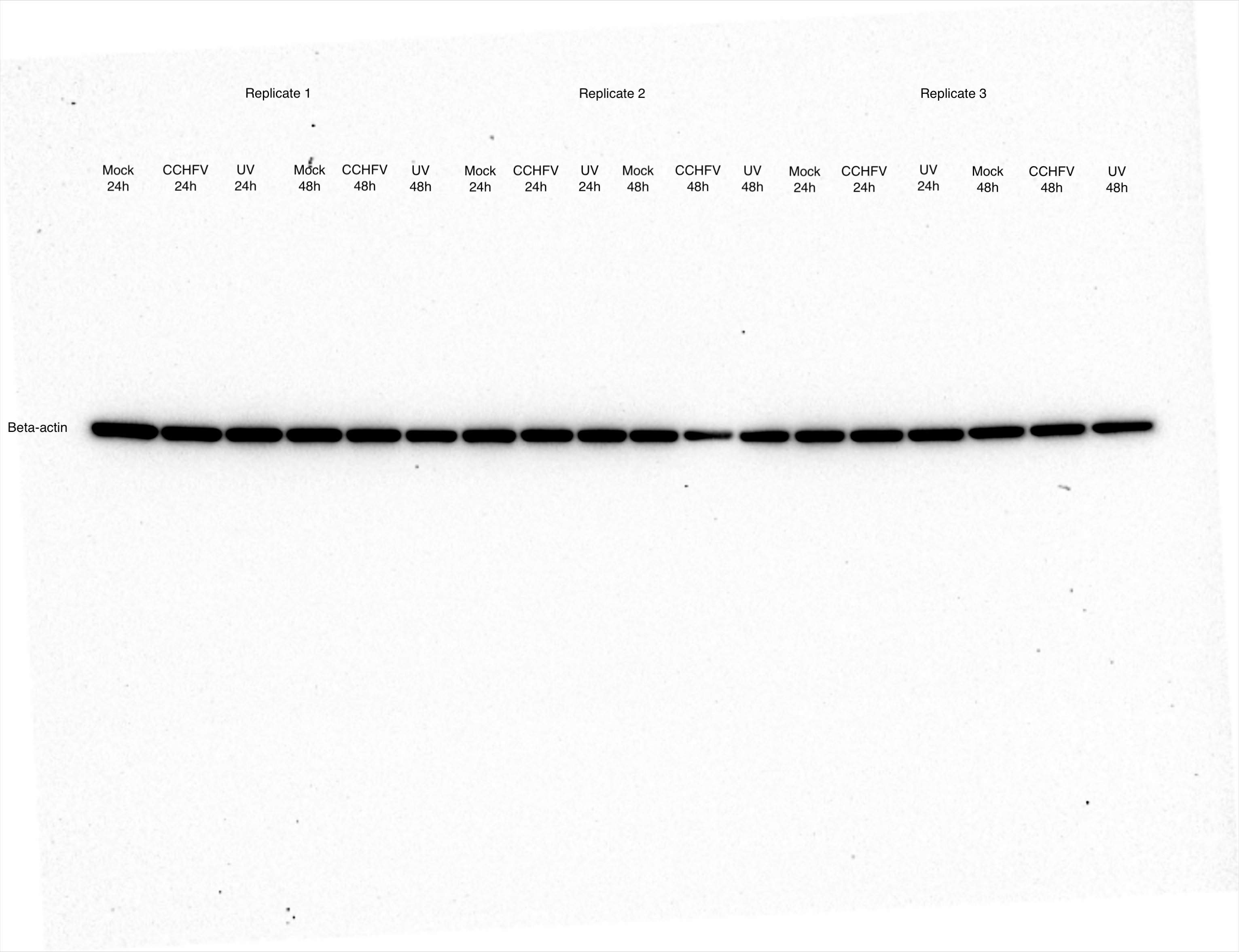

Supplement: Figure 5—source data 1. [file elife-76071-fig5-data1.zip › Source_Data_1/WB_CCHFV_ISG/WB Images copy/Nprotein_Replicate1_2_3/Actin_300s_Final.tif]

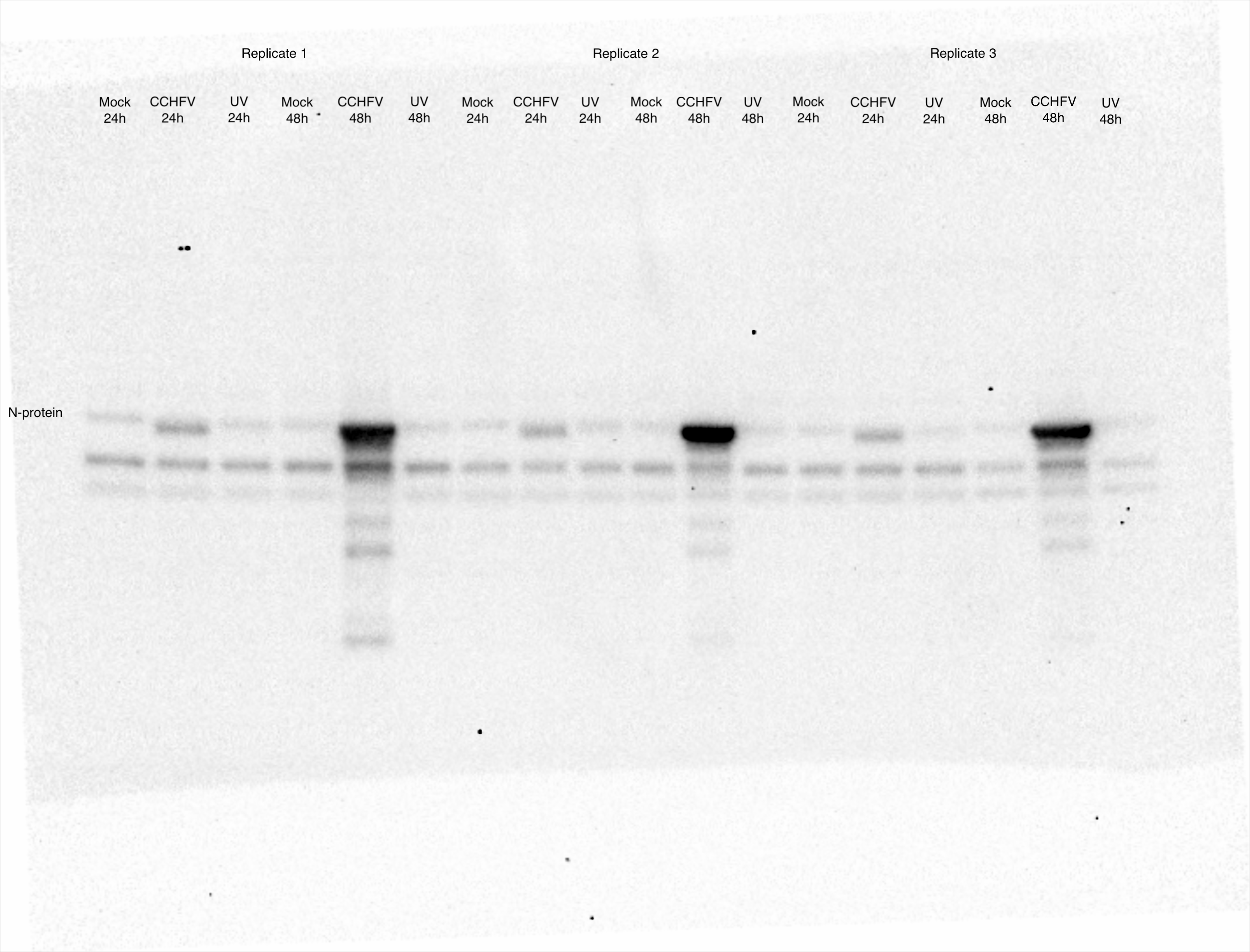

Supplement: Figure 5—source data 1. [file elife-76071-fig5-data1.zip › Source_Data_1/WB_CCHFV_ISG/WB Images copy/Nprotein_Replicate1_2_3/NP_Redev_60s_Final.tif]

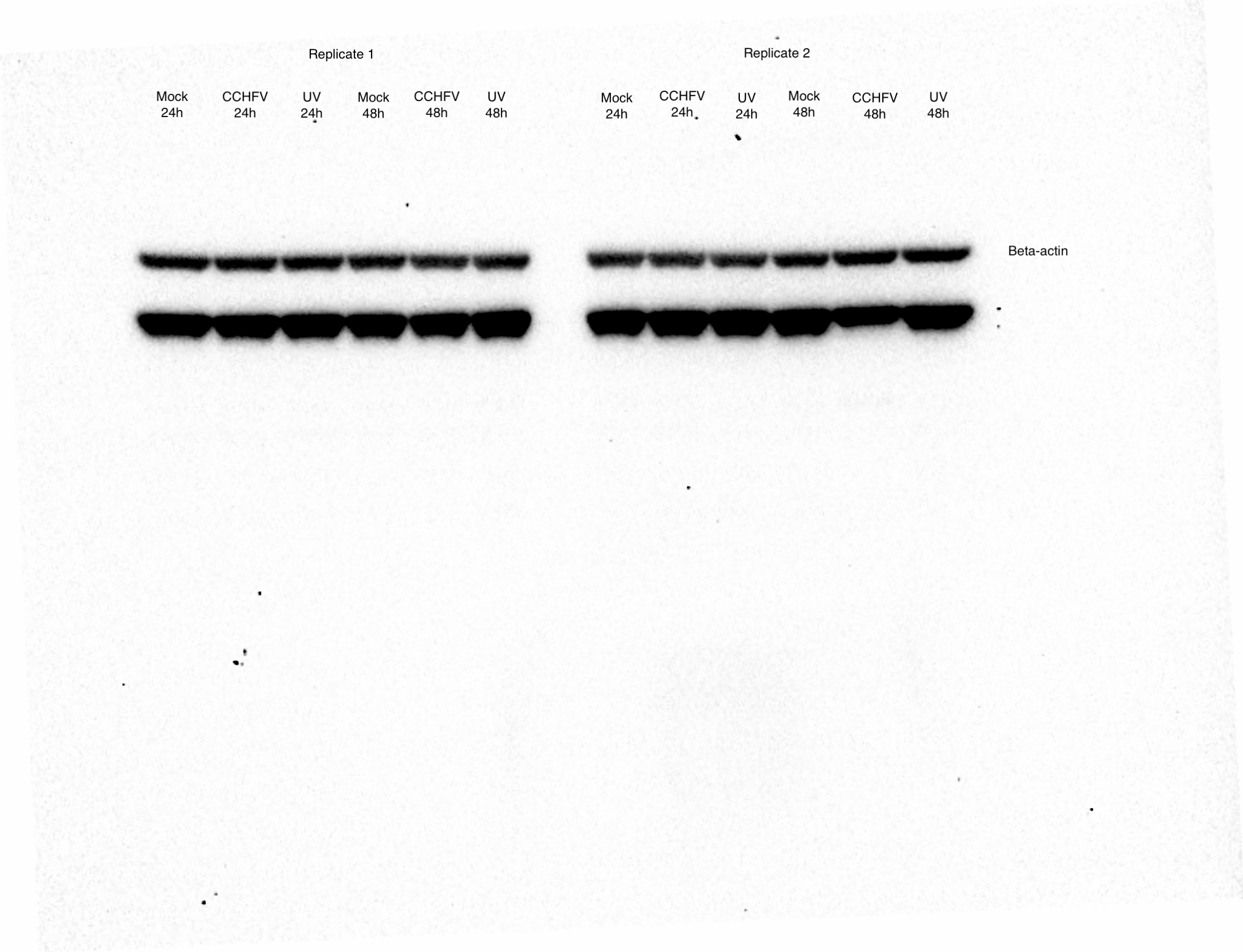

Supplement: Figure 5—source data 1. [file elife-76071-fig5-data1.zip › Source_Data_1/WB_CCHFV_ISG/WB Images copy/Replicate1_Replicate2/actin_120s_Gel2_Final.tif]

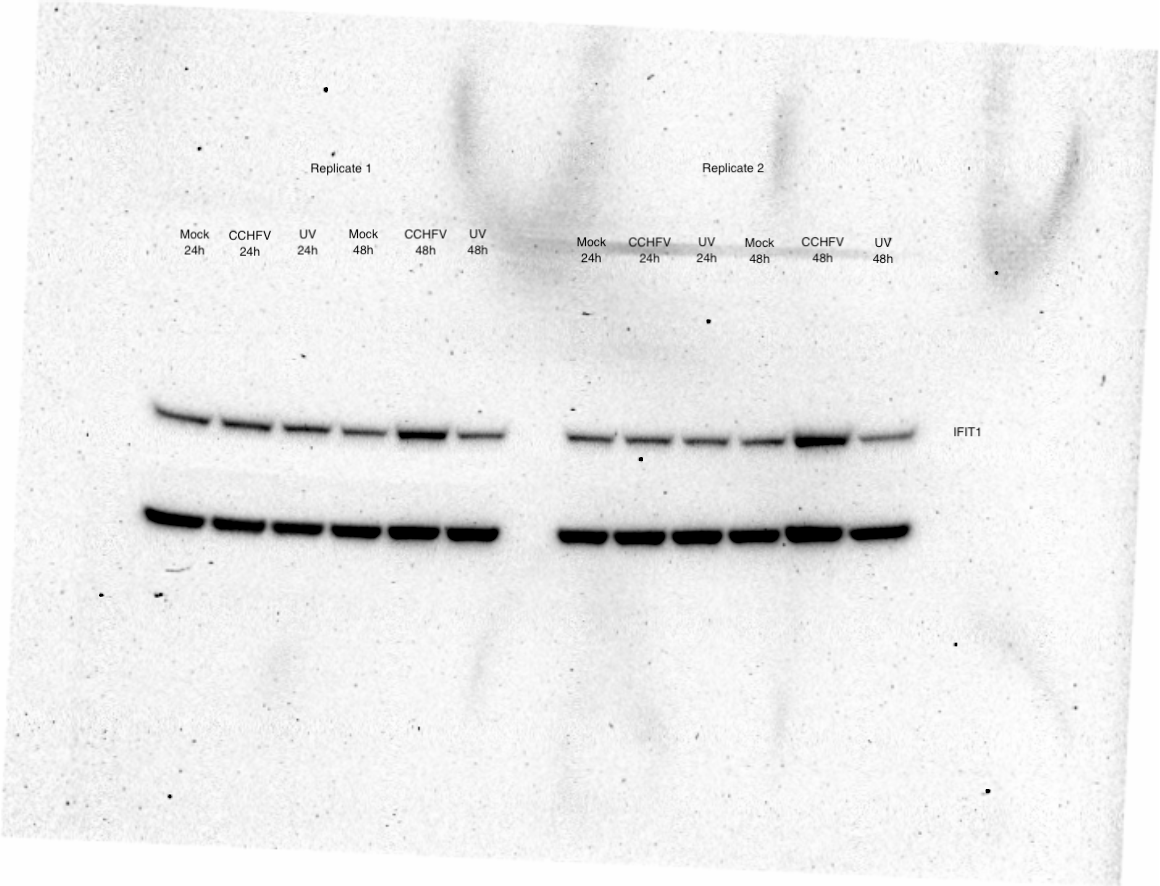

Supplement: Figure 5—source data 1. [file elife-76071-fig5-data1.zip › Source_Data_1/WB_CCHFV_ISG/WB Images copy/Replicate1_Replicate2/IFIT1_1200s_Final.tif]

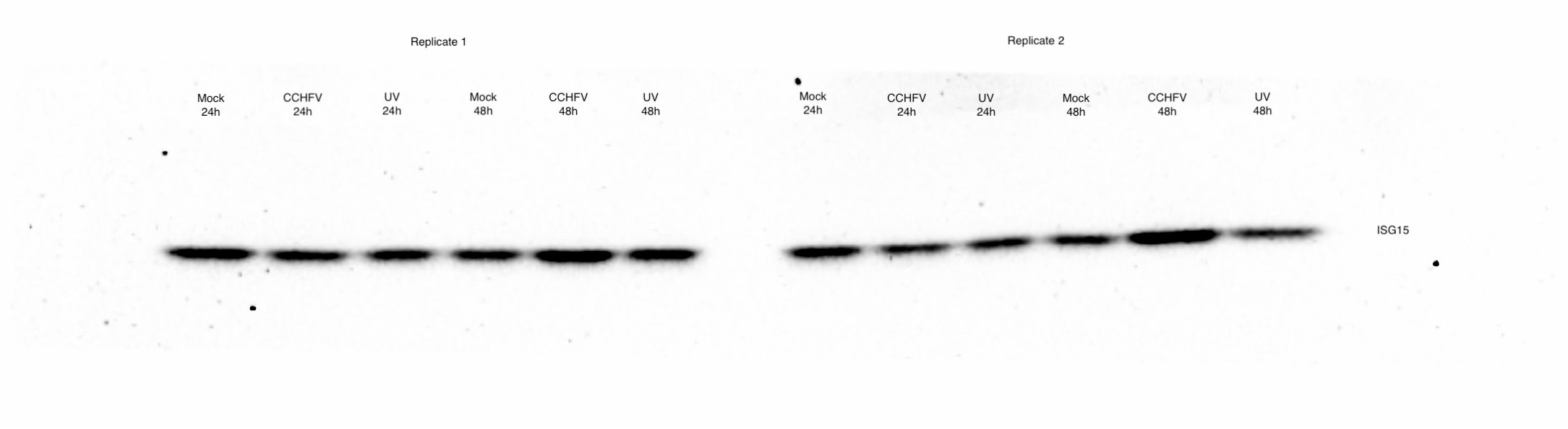

Supplement: Figure 5—source data 1. [file elife-76071-fig5-data1.zip › Source_Data_1/WB_CCHFV_ISG/WB Images copy/Replicate1_Replicate2/ISG15_1200s_R1_Final.tif]

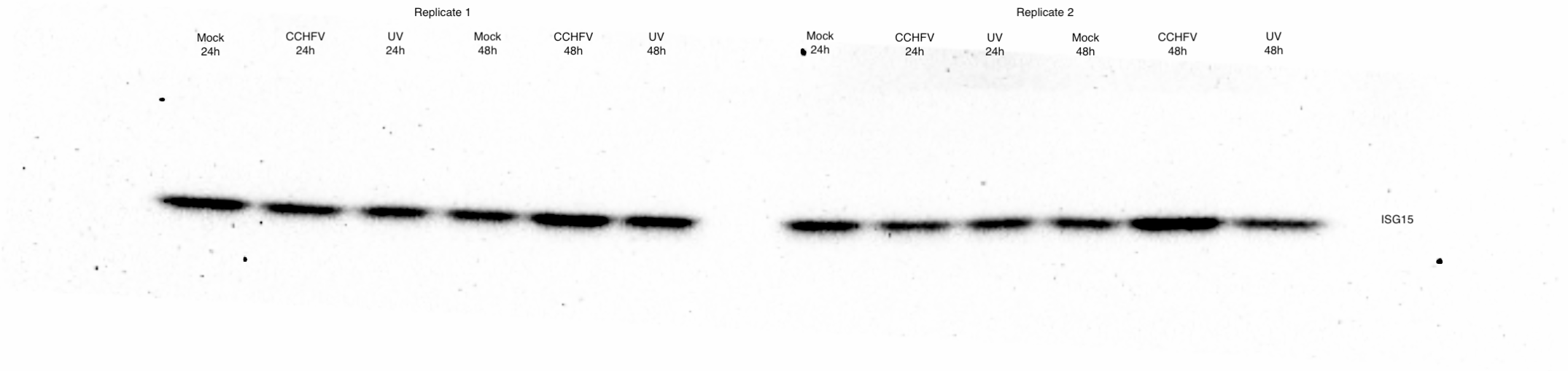

Supplement: Figure 5—source data 1. [file elife-76071-fig5-data1.zip › Source_Data_1/WB_CCHFV_ISG/WB Images copy/Replicate1_Replicate2/ISG15_1200s_R2_Final.tif]

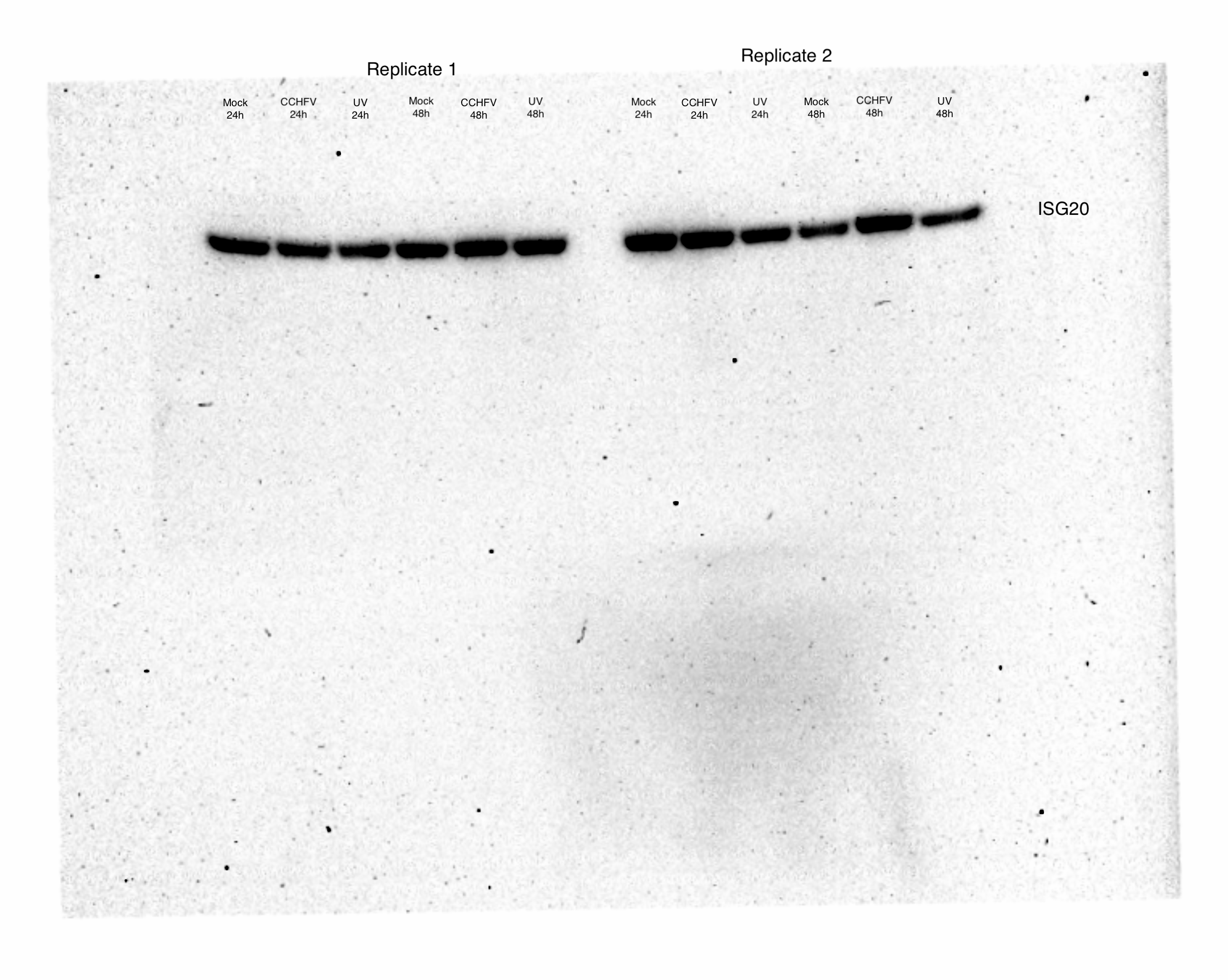

Supplement: Figure 5—source data 1. [file elife-76071-fig5-data1.zip › Source_Data_1/WB_CCHFV_ISG/WB Images copy/Replicate1_Replicate2/ISG20_1200s_R1_Final.tif]

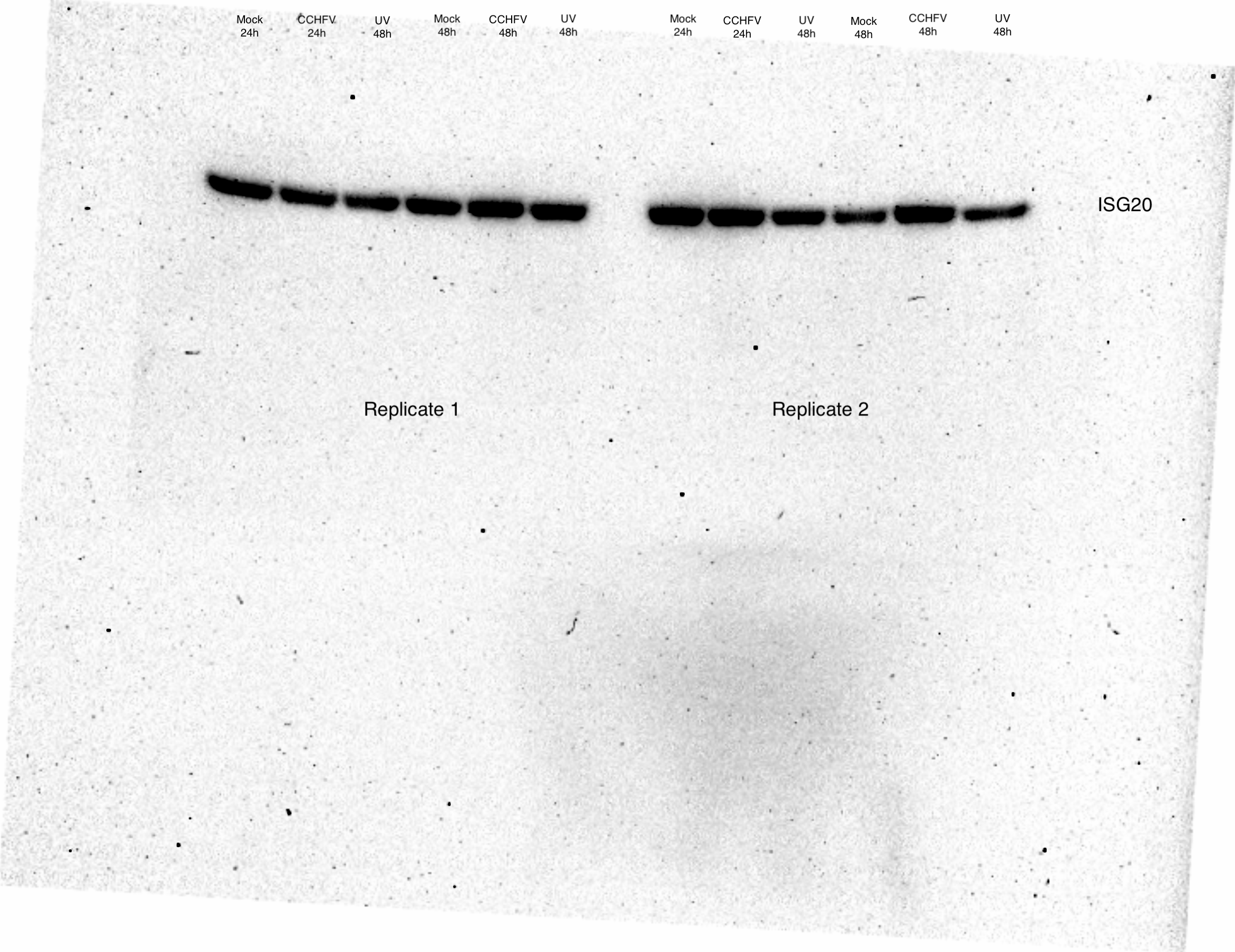

Supplement: Figure 5—source data 1. [file elife-76071-fig5-data1.zip › Source_Data_1/WB_CCHFV_ISG/WB Images copy/Replicate1_Replicate2/ISG20_1200s_R2_Final.tif]

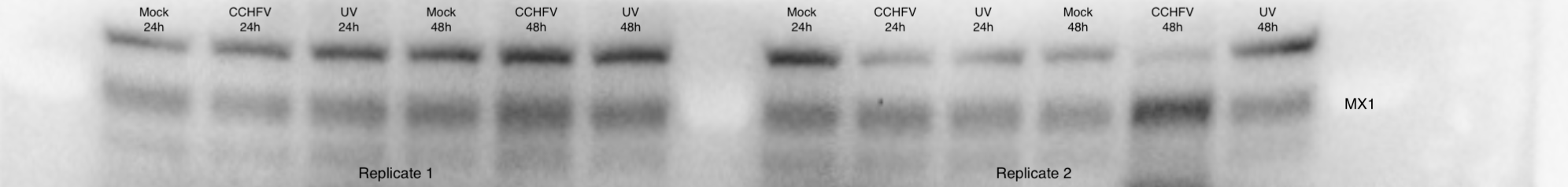

Supplement: Figure 5—source data 1. [file elife-76071-fig5-data1.zip › Source_Data_1/WB_CCHFV_ISG/WB Images copy/Replicate1_Replicate2/MX1_SELECT_180s_Final.tif]

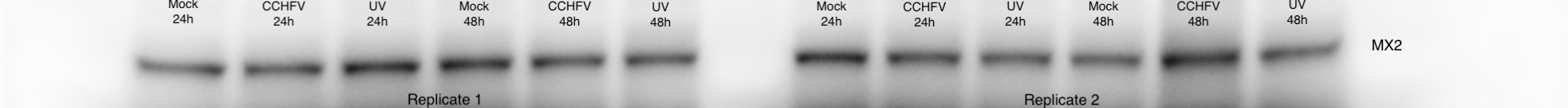

Supplement: Figure 5—source data 1. [file elife-76071-fig5-data1.zip › Source_Data_1/WB_CCHFV_ISG/WB Images copy/Replicate1_Replicate2/MX2_SELECT_90s_Final.tif]

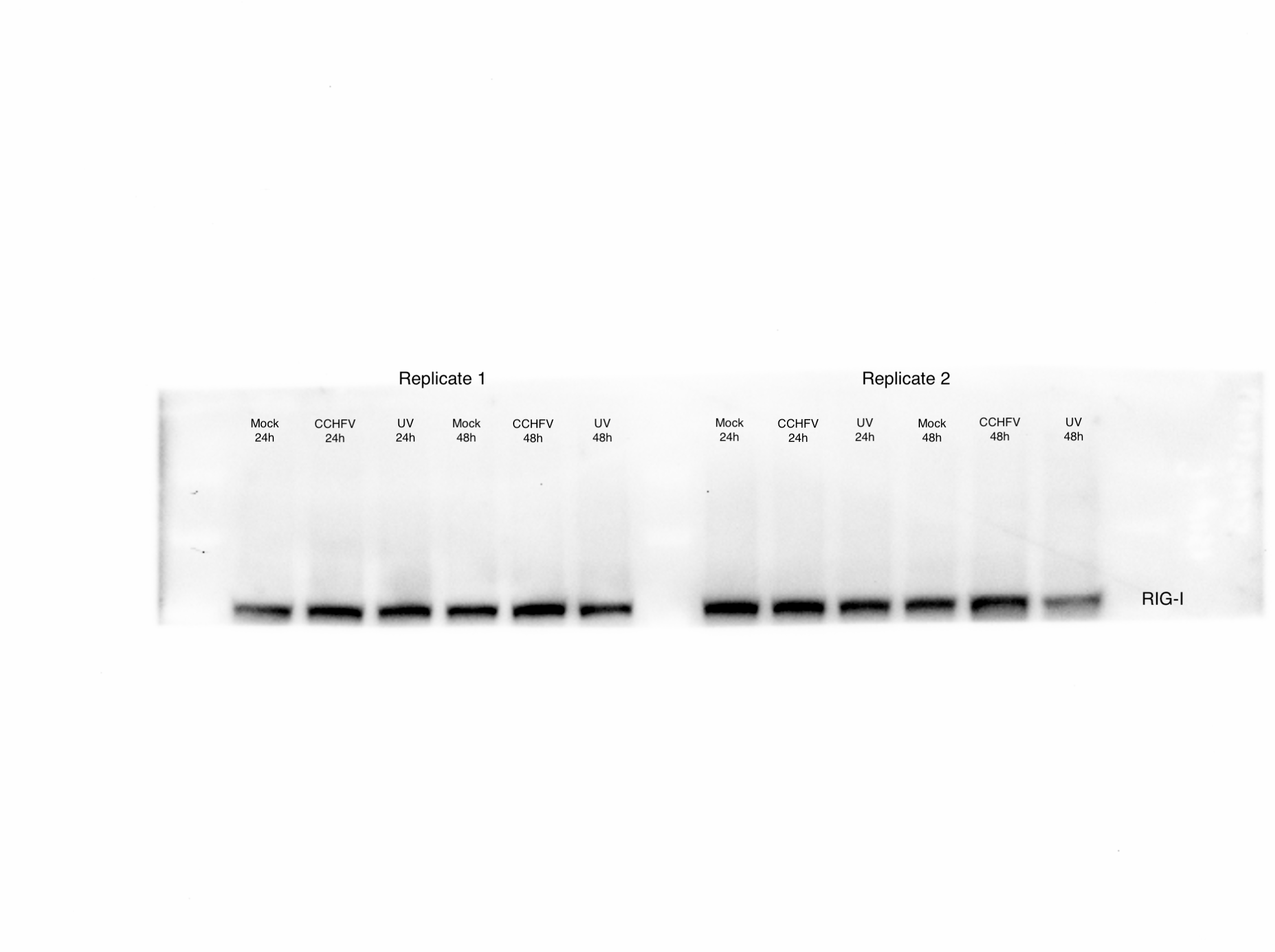

Supplement: Figure 5—source data 1. [file elife-76071-fig5-data1.zip › Source_Data_1/WB_CCHFV_ISG/WB Images copy/Replicate1_Replicate2/RIGI_select_180s_Final.tif]

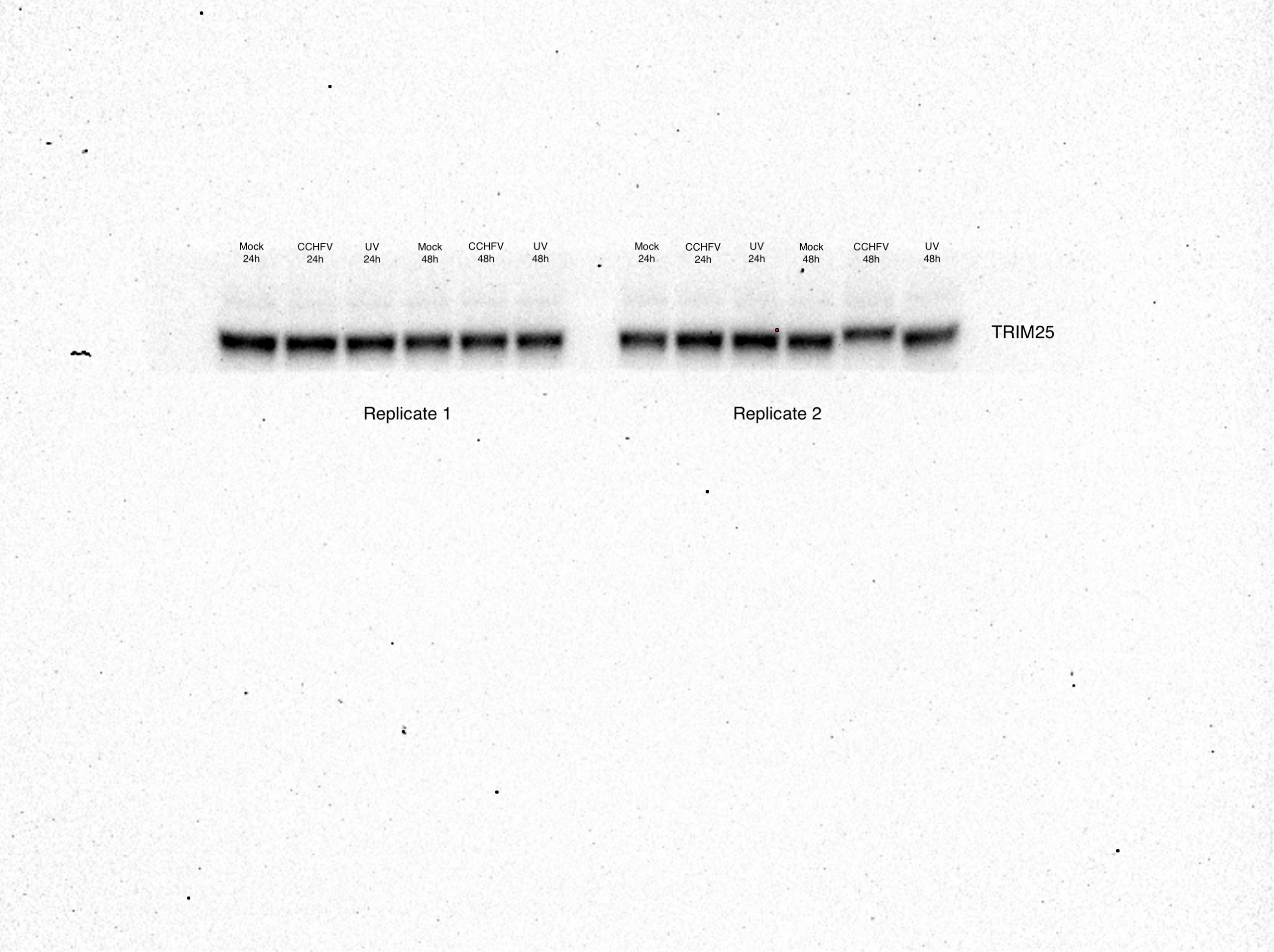

Supplement: Figure 5—source data 1. [file elife-76071-fig5-data1.zip › Source_Data_1/WB_CCHFV_ISG/WB Images copy/Replicate1_Replicate2/TRIM25_300s_Final.tif]

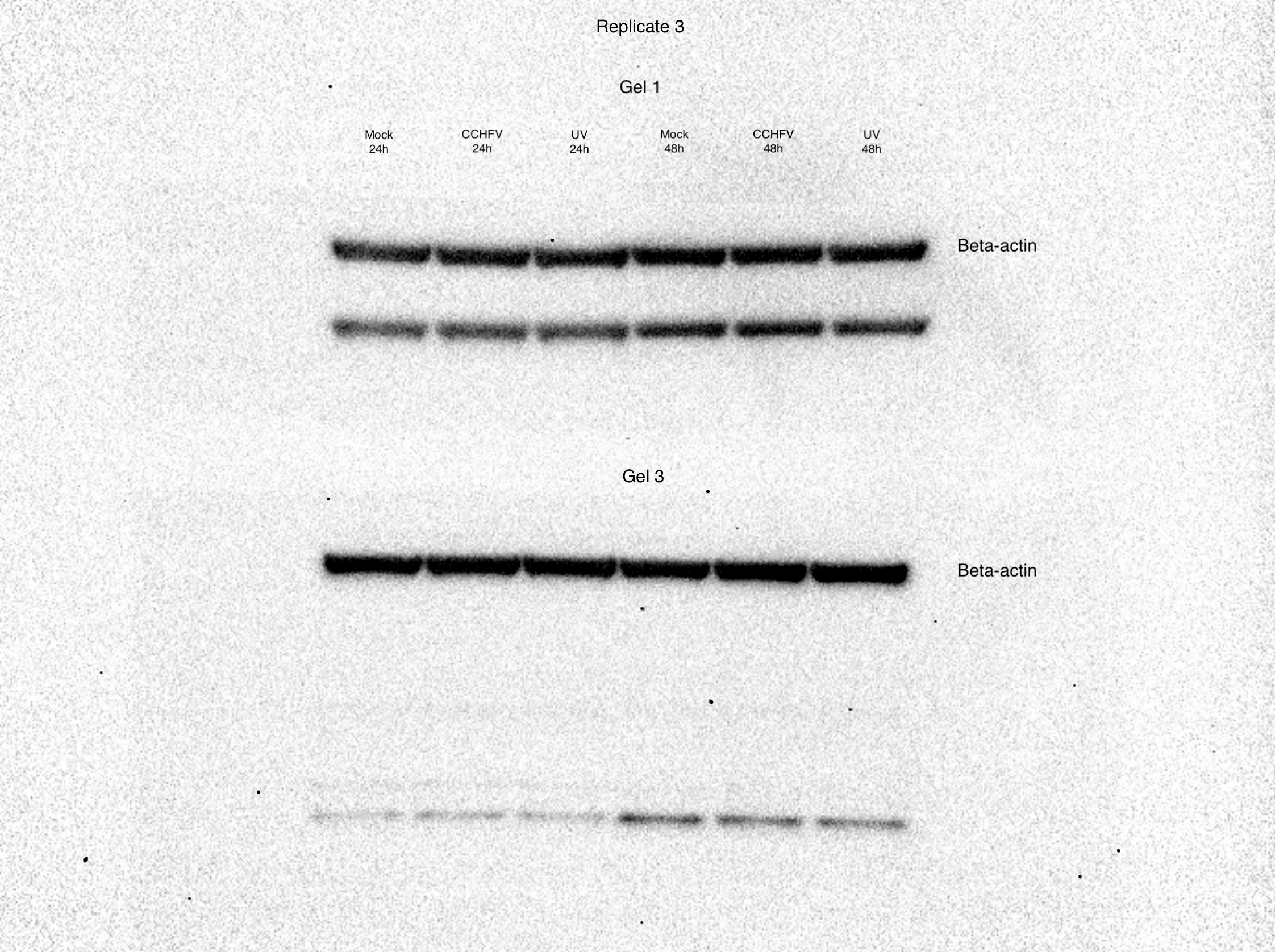

Supplement: Figure 5—source data 1. [file elife-76071-fig5-data1.zip › Source_Data_1/WB_CCHFV_ISG/WB Images copy/Replicate3/Actin_60s_Gel1_Gel3_Final copy.tif]

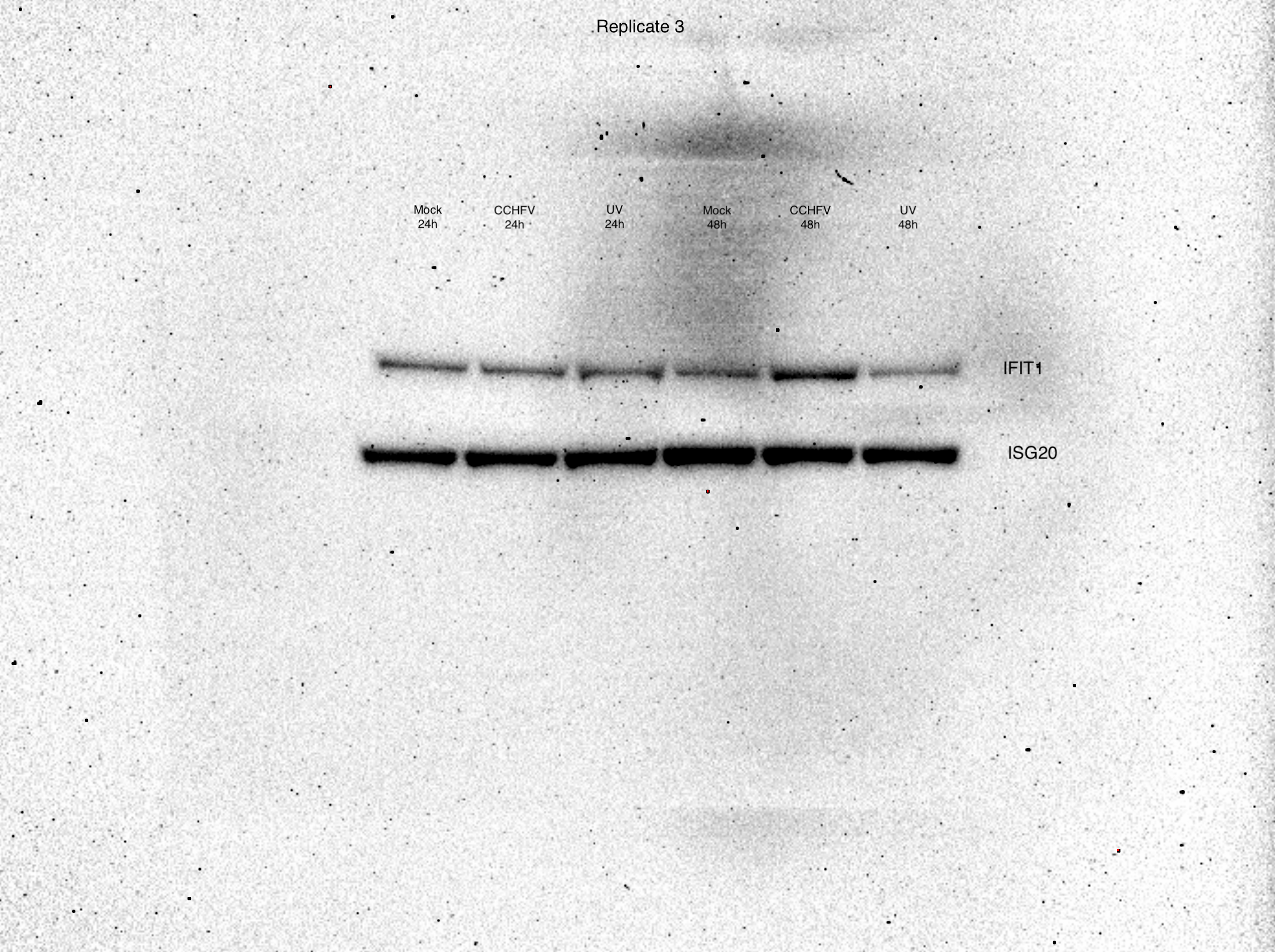

Supplement: Figure 5—source data 1. [file elife-76071-fig5-data1.zip › Source_Data_1/WB_CCHFV_ISG/WB Images copy/Replicate3/IFIT1_ISG20_1200s_Final.tif]

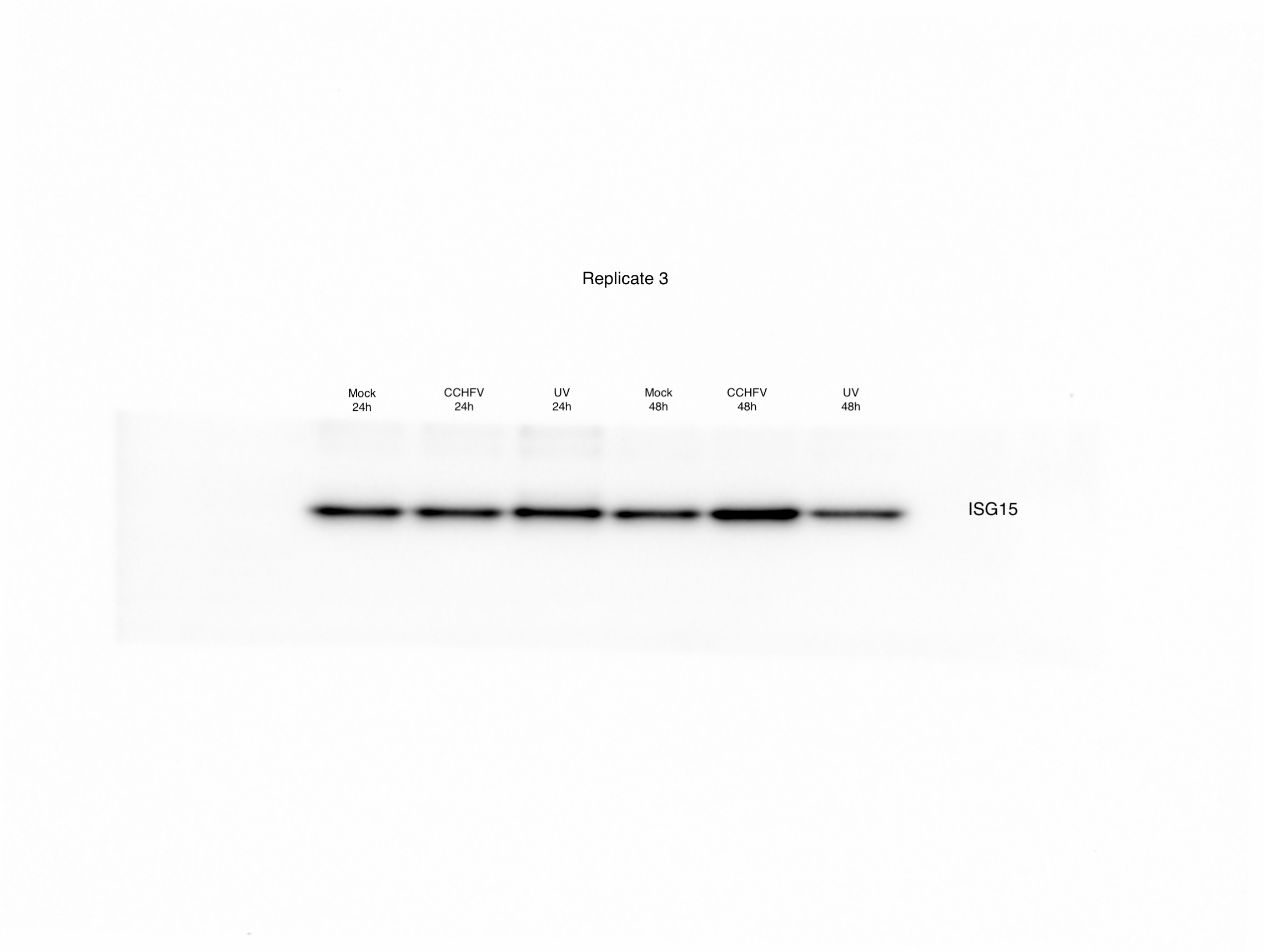

Supplement: Figure 5—source data 1. [file elife-76071-fig5-data1.zip › Source_Data_1/WB_CCHFV_ISG/WB Images copy/Replicate3/ISG15_Select_20s_Final.tif]

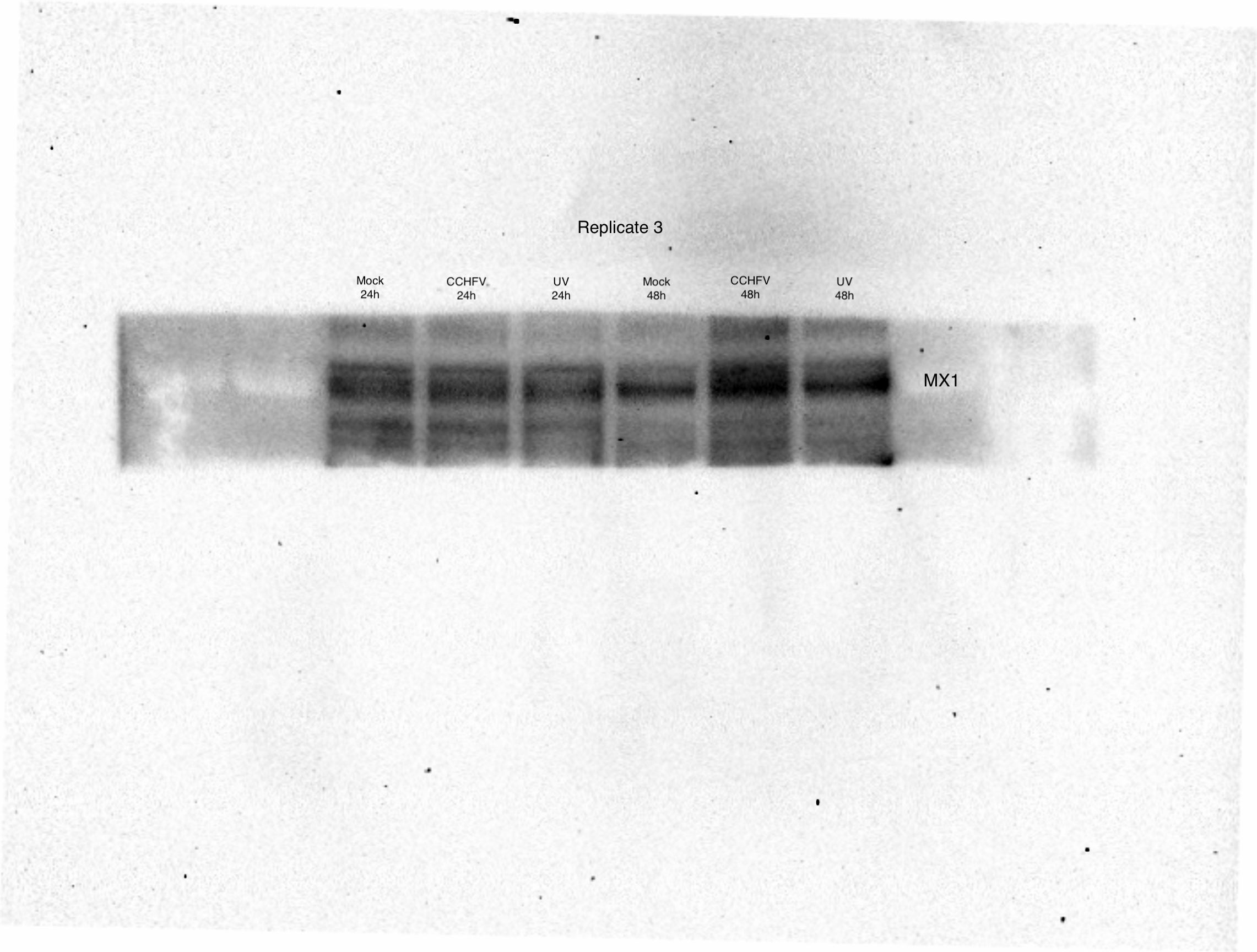

Supplement: Figure 5—source data 1. [file elife-76071-fig5-data1.zip › Source_Data_1/WB_CCHFV_ISG/WB Images copy/Replicate3/MX1_Select_300s_Final.tif]

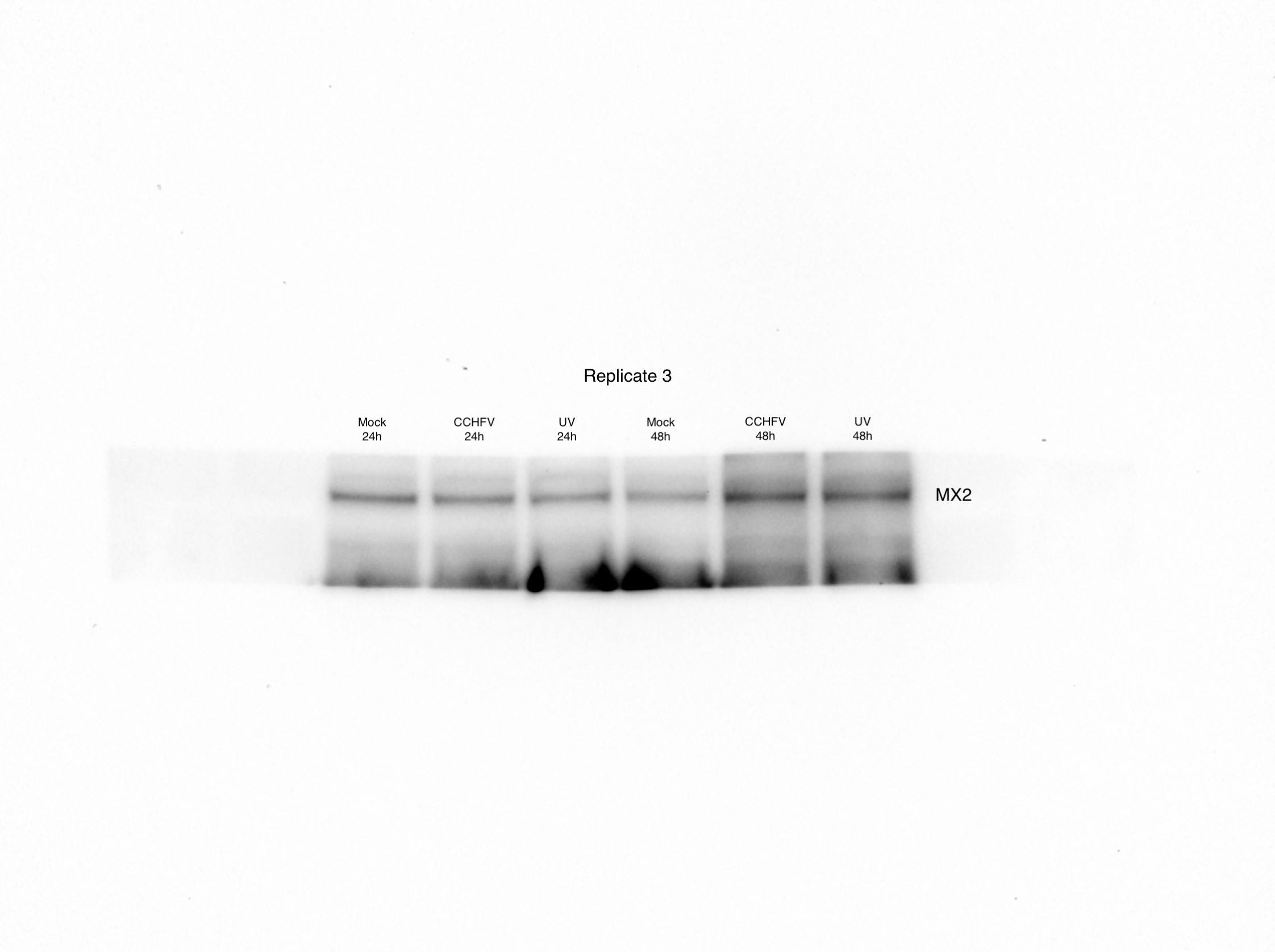

Supplement: Figure 5—source data 1. [file elife-76071-fig5-data1.zip › Source_Data_1/WB_CCHFV_ISG/WB Images copy/Replicate3/MX2_Select_60s_Final.tif]

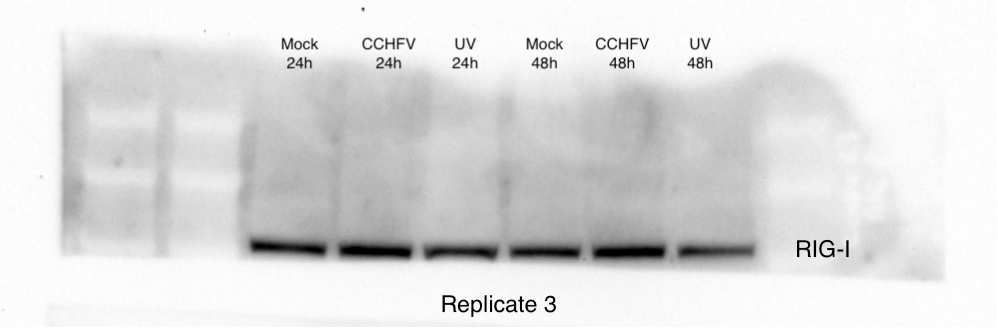

Supplement: Figure 5—source data 1. [file elife-76071-fig5-data1.zip › Source_Data_1/WB_CCHFV_ISG/WB Images copy/Replicate3/RIG_select_120s_Final.tif]

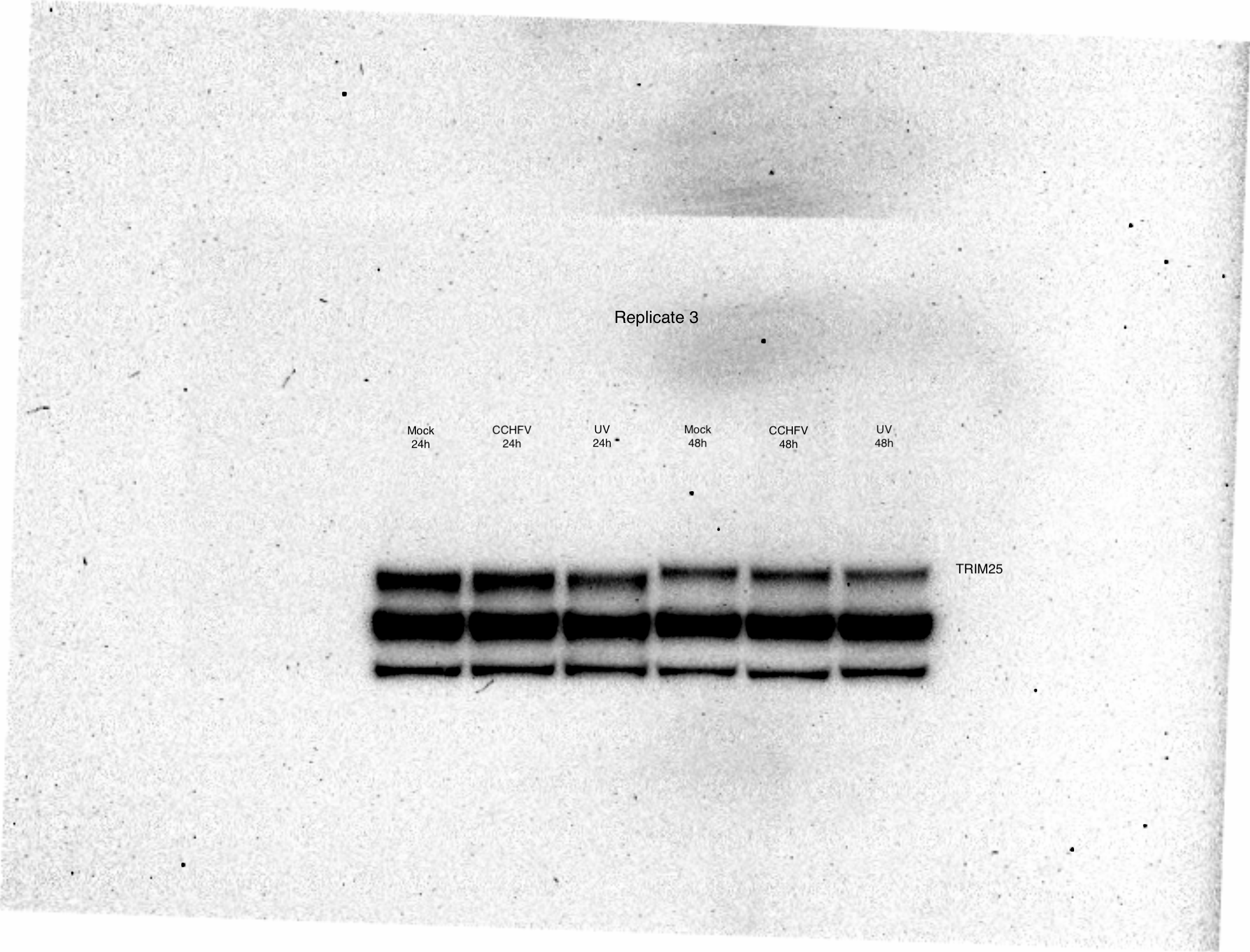

Supplement: Figure 5—source data 1. [file elife-76071-fig5-data1.zip › Source_Data_1/WB_CCHFV_ISG/WB Images copy/Replicate3/TRIM25_1200s_Final.tif]
